# Persistierende Proktitis und anorektales Ulkus

**DOI:** 10.1007/s00104-023-01961-3

**Published:** 2023-09-04

**Authors:** Katharina Tripolt-Droschl, Sarah Schwarz, Hannes Schmölzer, Anna Schiefer-Niederkorn, Birgit Sadoghi

**Affiliations:** 1 Department of Dermatology and Venereology, Medical University of Graz, Auenbruggerplatz 8, 8036 Graz, Österreich; 2 Department of Surgery, Landesklinikum Graz II, Göstingerstr. 22, 8020 Graz, Österreich

## Anamnese und Klinik

Im Oktober 2019 stellte sich ein 39-jähriger männlicher Patient mit proktitischen Beschwerden, einem analen Ulkus und dem Verdacht auf eine neuerliche Syphilis-Infektion an einer Abteilung für Infektiologie vor. Zuletzt war der Patient im März lege artis aufgrund einer Syphilis-Infektion mit Benzathin-Benzylpenicillin behandelt worden. Der Patient identifizierte sich als MSM (Mann, welcher Sex mit Männern hat), war seit 2006 HIV-positiv und erhielt zum Zeitpunkt der Vorstellung eine antiretrovirale Therapie mit Emtricitabin/Tenofoviralafenamid/Rilpivirin 200 mg/25 mg/25 mg. Die CD4/CD8-Ratio betrug 0,80. Nach Aufklärung und auch auf Wunsch des Patienten wurde aufgrund der neuerlichen Beschwerdesymptomatik 3‑mal Benzathin-Benzylpenicillin 2,4 Mio. IE im Abstand je einer Woche verabreicht (die Syphilis-Serologie ergab damals RPR 1:1, IgM-ELISA grenzwertig positiv, Treponema-pallidum-Partikel-Agglutinationstest (TPPA) > 1:1280, Treponema pallidum membran protein Test (TMPA)-Screen positiv).

**Figure 1:**
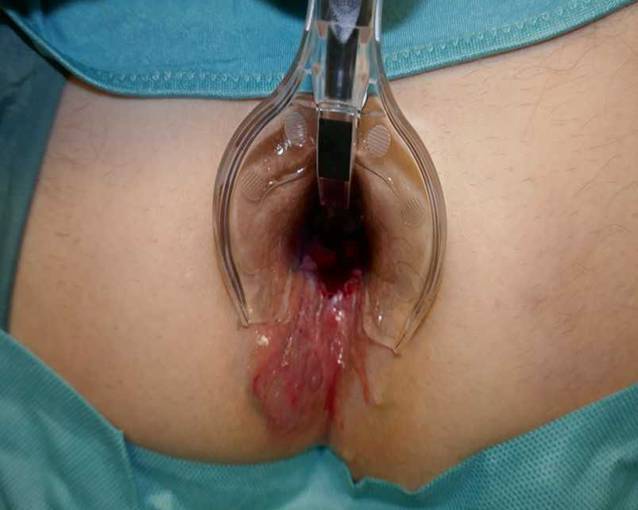

**Figure 2:**
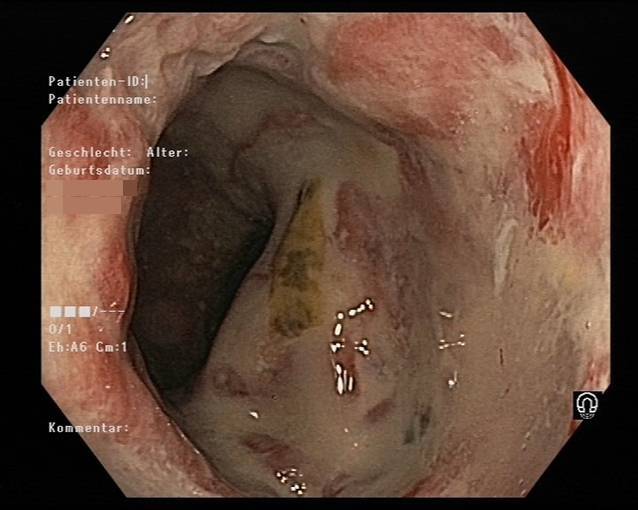

**Figure 3:**
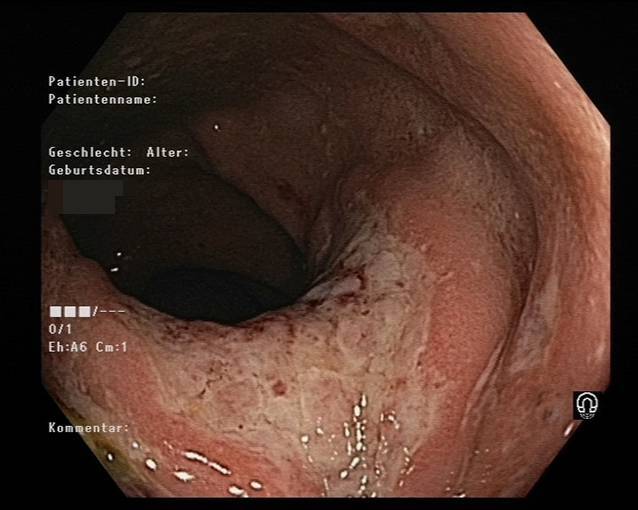

Das Ulkus war dennoch größenprogredient, und eine daraufhin im November 2019 durchgeführte Magnetresonanztomographie (MRT) zeigte eine Ausdehnung in den M. sphincter ani externus, M. sphincter ani internus und M. levator ani sowie einen sich am dorsalen Ende des Ulkus befindlichen Abszess mit einem Durchmesser von 3,5 × 1,5 × 3,4 cm. Außerdem wurden mehrere reaktive inguinale Lymphknoten festgestellt. Der Patient wurde folglich an der Abteilung für Chirurgie (Abb. 1) vorgestellt. Im November 2019 wurde der Abszess drainiert, und im Zuge dessen wurden mehrere Stanzbiopsien entnommen. Die Histologie ergab keinen Hinweis für Malignität, dennoch wurde eine hochgradige chronische Entzündung mit neutrophilen Granulozyten, Blut, Fibrin und Granulationsgewebe beschrieben. Da es weiterhin zu keiner Abheilung des Ulkus kam, wurde ein erneuter chirurgischer Eingriff vorgeschlagen, den der Patient im Februar 2020 allerdings aus Angst vor einer möglichen Inkontinenz ablehnte. Des Weiteren führte man sowohl im Juni als auch im Juli 2020 Koloskopien (Abb. 2 und 3) durch, bei denen histologisch erneut Granulationsgewebe und eine chronische Entzündung festgestellt wurden.

## Weiterer Verlauf

Im weiteren Verlauf wurde der Patient aufgrund der Persistenz des Ulkus und der suszipierten sexuell übertragbaren Erkrankung im Juli 2020 an die Ambulanz für sexuell übertragbare Erkrankungen an die Universitätsklinik für Dermatologie und Venerologie überwiesen. Er stellte sich mit einem ca. 2 cm großen, sichtbaren, sich in den Analkanal ausdehnenden Ulkus vor. Eine Multiplex-PCR (Euro Array STI 11, Euroimmun® Lübeck, Deutschland) wurde an allen relevanten Stellen (oral, urethral, rektal) durchgeführt.

## Wie lautet Ihre Diagnose?

## Erklärung

Aufgrund der Begleitsymptomatik (persistierende Proktitis, Lymphadenopathie), der sexuellen Vorgeschichte des Patienten (MSM mit wechselnden Sexualpartnern und ungeschütztem Analverkehr) und des fehlenden Ansprechens auf die Syphilisbehandlung wurde die Verdachtsdiagnose eines Lymphogranuloma venereum gestellt.

## Weitere Diagnostik und Therapie

*Chlamydia trachomatis* (CT) konnte im Rektalbereich mittels Multiplex-PCR nachgewiesen werden. Die konsekutive Genotypisierung bestätigte die Diagnose LGV (CT Serovar L2). Andere sexuell übertragbare Infektionen einschließlich Gonorrhö, Syphilis, Trichomoniasis und Ulcus molle konnten ausgeschlossen werden. Der Patient war gegen Hepatitis B immunisiert und die Hepatitis-C-Serologie zeigte sich ebenfalls negativ. Daraufhin wurde Doxycyclin 100 mg 2‑mal täglich per os für 21 Tage verschrieben. Bei einer Kontrolle im September 2020 fiel der „Test-of-Cure“ auf *Chlamydia trachomatis* negativ aus, und im Zeitraum von wenigen Wochen kam es zur vollständigen Rückbildung der klinischen Symptomatik des Patienten.

## Hintergrundinformationen zum Krankheitsbild

### Lymphogranuloma venereum

LGV ist eine sexuell übertragbare Infektion, die durch invasive Varianten von *Chlamydia trachomatis* Serovare L1, L2 und L3 verursacht wird [1]. Die Inzidenz ist weltweit steigend [1, 2]. Im Jahr 2019 wurden 3112 Fälle von LGV in der Europäischen Union/dem Europäischen Wirtschaftsraum (EU/EWR) gemeldet, von denen nur 10 Fälle bei Frauen nachgewiesen wurden [2].

**Diagnose:** Lymphogranuloma venereum (LGV)

LGV verläuft in 3 Stadien, beginnend mit einer Papel, aus welcher 3 bis 30 Tage nach der Infektion ein Ulkus entsteht, typischerweise gefolgt von einer schmerzhaften einseitigen inguinalen Lymphadenopathie [1]. Im weiteren Verlauf kann es bei der Erkrankung zur Proktokolitis, zur Bildung von Fisteln, Abszessen und Strikturen kommen. Wenn LGV über Monate bis Jahre unbehandelt bleibt, kann es auch zu irreversiblen Lymphödemen kommen [1]. Außerdem leiden die Patienten häufig unter systemischen Symptomen wie Fieber, Schüttelfrost und Lymphadenitis [1]. Bei bis zu einem Viertel der Patienten treten jedoch die oben genannten typischen Symptome nicht auf [1].

Die PCR auf *Chlamydia trachomatis* und die anschließende Untersuchung des Genotyps ist die einzig mögliche Methode zum Nachweis eines LGV. In mehreren Leitlinien wird Doxycyclin 100 mg 2‑mal täglich per os für 21 Tage als Erstlinientherapie empfohlen. Als Zweit- und Drittlinientherapie haben sich Erythromycin oder Azithromycin bewährt [1].

### Proktitis

Die Proktitis präsentiert sich als Entzündung der analen und rektalen Schleimhaut, die eine Vielzahl an Ursachen haben kann, darunter beispielsweise chronisch entzündliche Darmerkrankungen (CED), Strahlentherapie, chemische Einwirkungen, Traumata, Malignome, wie z. B. Lymphome, sowie Infektionen [3]. Eine infektiöse Proktitis oder Proktokolitis wird durch nichtvenerische Erreger wie *Escherichia coli, Shigella spp., Campylobacter spp.* und *Clostridium difficile* verursacht [3]. Vor allem bei MSM sind sexuell übertragbare Infektionen wie Gonorrhö und im Falle einer ulzerativen Proktitis Herpes genitalis, primäre Syphilis, Ulcus molle, Granuloma inguinale und Lymphogranuloma venereum (LGV) wichtige Differenzialdiagnosen [3]. Zu den typischen Symptomen einer Proktitis gehören rektale Blutungen, Tenesmen (häufiger Stuhldrang), schleimiger Ausfluss, Obstipation oder Diarrhö.

Seltenere Ursachen für anorektale Abszesse sind abdominelle oder pelvine Infektionen wie Divertikulitis, perforierende Karzinome, Morbus Crohn und LGV. Außerdem können anorektale Fisteln aus einem Abszess, aber auch spontan oder als postoperative Komplikation entstehen [1, 5]. Das anorektale LGV kann somit Krankheiten wie CED oder andere Infektionskrankheiten im Bereich des Anorektums vortäuschen. Aufgrund der Ähnlichkeit der Symptomatik wird das LGV daher oftmals falsch oder mit erheblicher Verzögerung diagnostiziert, wenngleich es eine heilbare Erkrankung darstellt [1, 4, 5].

## Fazit für die Praxis

- Bei anorektalen und/oder proktitischen Symptomen sollte eine ausführliche Sexualanamnese erhoben werden, einschließlich früherer sexuell übertragbarer Infektionen (der Patientin/des Patienten selbst und ihrer/seiner Partnerinnen/Partner), der sexuellen Orientierung und des Risikoverhaltens (z. B. Anzahl der Sexualpartnerinnen/Sexualpartner, Verwendung von Kondomen, Analverkehr).
- Insbesondere Patienten, die sich als MSM identifizieren und an Proktitis/Proktokolitis leiden, sollten rektal auf *Chlamydia trachomatis* getestet werden.
